# Arrhythmogenic left ventricular cardiomyopathy managed with CRT-D: A case report

**DOI:** 10.1097/MD.0000000000047481

**Published:** 2026-03-06

**Authors:** Xinhe Cheng, Xin Wang, Yang Liu

**Affiliations:** a Department of Infectious Diseases, Gansu Provincial Hospital, Lanzhou, China; b The First School of Clinical Medicine, Lanzhou University, Lanzhou, China; c Allergy Disease Diagnosis and Treatment Center, Gansu Provincial Hospital, Lanzhou, China.

**Keywords:** arrhythmogenic left ventricular cardiomyopathy, implantable cardioverter-defibrillator, magnetic resonance imaging, myocardial fibrosis, ventricular tachycardia

## Abstract

**Rationale:**

Arrhythmogenic left ventricular cardiomyopathy (ALVC) is an arrhythmogenic cardiomyopathy characterized by nonischemic left-ventricular scar, ventricular arrhythmias, and risk of sudden cardiac death. Diagnosis is often guided by cardiac magnetic resonance (CMR) and clinical criteria.

**Patient concerns:**

A 51-year-old man presented with 10 months of intermittent chest tightness and dyspnea, which worsened over 1 month, and 1 episode of syncope.

**Diagnoses:**

Transthoracic echocardiography showed severe left ventricular systolic dysfunction (left ventricular ejection fraction 19%). CMR demonstrated nonischemic subepicardial fibrosis and diffuse left ventricular free-wall thinning consistent with ALVC. Coronary angiography revealed no significant stenosis. A clinical cardiomyopathy panel test reported findings supportive of ALVC.

**Interventions:**

Guideline-directed medical therapy was initiated, and a cardiac resynchronization therapy defibrillator was implanted for left ventricular dysfunction with dyssynchrony and arrhythmic risk.

**Outcomes:**

Ten days after discharge, a localized device-pocket infection occurred and was managed with debridement, negative-pressure therapy, and intravenous vancomycin, with full recovery. At 3 and 6 months, left ventricular ejection fraction improved to 27% and 36%, respectively, and device interrogation documented no malignant ventricular arrhythmias.

**Lessons:**

This case underscores the diagnostic value of CMR and supports the combined strategy of cardiac resynchronization therapy defibrillator in left ventricular–dominant disease with dyssynchrony, aligning with contemporary guidelines.

## 1. Introduction

Arrhythmogenic left ventricular cardiomyopathy (ALVC), also known as left ventricular involvement cardiomyopathy, tumorigenic left ventricular cardiomyopathy, and desmoplakin cardiomyopathy,^[1–4]^ typically presents with nonspecific symptoms such as chest tightness, dyspnea, syncope, arrhythmia, heart failure, or features mimicking dilated cardiomyopathy (DCM).^[5,6]^ This disease affects both the electrophysiological and mechanical functions of the heart, predisposing patients to life-threatening arrhythmias and sudden cardiac death (SCD). Due to its rarity and heterogenous clinical presentations, diagnosing ALVC remains challenge. A lack of awareness often delays timely treatment. Herein, we report a case of ALVC in a male patient to provide clinical reference for improved diagnosis and treatment strategies.

## 2. Case presentation

A 51-year-old man was admitted on June 1, 2023, with a 10-month history of intermittent chest tightness and dyspnea, which had worsened over the preceding month. His symptoms initially occurred during physical exertion, such as climbing slopes or stairs, and typically resolved within 3 to 5 minutes of rest, without associated chest pain, palpitations, cough, or sputum. Over the month before admission, dyspnea progressed to occur even during level walking. He also experienced 1 episode of syncope with transient loss of consciousness, without urinary or fecal incontinence, and recovered spontaneously within minutes. The timeline of events and treatment details are summarized in Table 1 and Table S1, Supplemental Digital Content, https://links.lww.com/MD/R389, respectively.

**Table 1:** Timeline of the case.

Date/period	Milestone	Details
August 2022	Symptom onset	Exertional chest tightness and dyspnea begin
June 1, 2023	Hospital admission	Initial evaluation; ECG/TTE/Chest X-ray; labs
June 2, 2023	Holter monitoring	Frequent PVCs; non VT runs
Jun 3, 2023	Coronary angiography	No significant stenosis
June (inpatient)	Cardiac MRI	LV free-wall subepicardial fibrosis; LV dilation/dysfunction
June (inpatient)	Genetic testing sent	Later confirms ALVC
June (inpatient)	CRT-D implantation	Biventricular pacing/defibrillation
June 19, 2023	Discharge	Clinically stable on GDMT
June 29, 2023	Pocket complication	Pain/erythema; debridement and negative-pressure drainage
July 6, 2023	Microbiology	Fungal spores and gram-positive cocci; IV vancomycin × 1 wk
September 2023	3-mo follow-up	LVEF 27%; no malignant ventricular arrhythmias
December 2023	6-mo follow-up	LVEF 36%; no malignant ventricular arrhythmias

ALVC = arrhythmogenic left ventricular cardiomyopathy, CRT-D = cardiac resynchronization therapy defibrillator, GDMT = guideline-directed medical therapy, ECG = electrocardiogram, LV = left ventricle, LVEF = left ventricle systolic dysfunction, PVCs = premature ventricular contractions, TTE = transthoracic echocardiography.

Initial evaluation at a local hospital showed markedly elevated NT-proBNP (16,814.5 pg/mL), with normal cardiac troponin I (<0.1 ng/mL) and creatine kinase-MB (1.95 ng/mL). Transthoracic echocardiography demonstrated left ventricular enlargement with global hypokinesia, pulmonary hypertension (estimated systolic pressure 60 mm Hg), mild pericardial effusion, and impaired left ventricular systolic and diastolic function, accompanied by significant mitral regurgitation and mild tricuspid regurgitation. Symptomatic treatment was initiated but failed to relieve symptoms, and the patient was subsequently referred to a tertiary center for further evaluation. Relevant history included smoking, alcohol consumption, and a maternal family history of syncope.

On physical examination, the patient was alert and in fair general condition. Vital signs showed a temperature of 36.1°C, heart rate of 66 bpm, respiratory rate of 19 bpm, and blood pressure of 87/64 mm Hg. Lung auscultation revealed coarse breath sounds with fine basal rales, without wheezes or pleural friction rubs. Cardiac examination demonstrated an irregular rhythm with normal heart sounds, without murmurs or pericardial friction rub. The abdomen was soft and nontender, with no hepatosplenomegaly, and no peripheral edema was observed.

**Figure 1.**
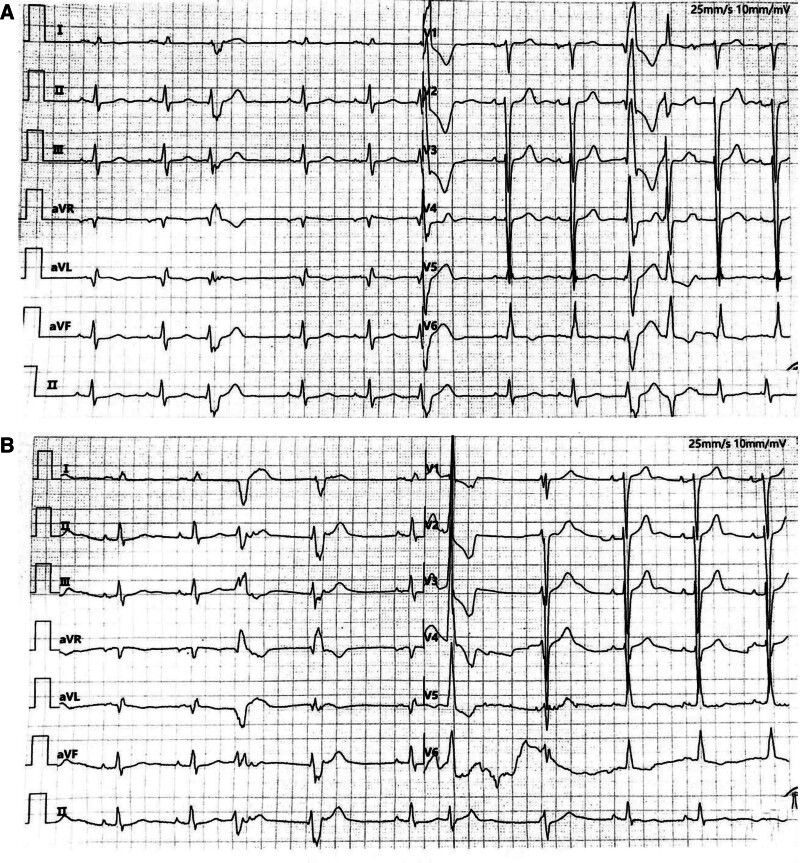
Admission ECG, bedside telemetry, and Holter summary (arrhythmia burden and baseline function). (A) admission ECG; (B) bedside monitoring ECG. ECG = electrocardiogram.

On admission, NT-proBNP was 3633 pg/mL and high-sensitivity cTnI was 0.009 ng/mL. ECG showed frequent premature ventricular contractions (PVCs), including couplets, poor R-wave progression in the right precordial leads, and ST–T abnormalities (Fig. 1A). Chest X-ray revealed cardiomegaly. Transthoracic echocardiography demonstrated severe left ventricular systolic dysfunction (LVEF 19%) with left ventricular enlargement, pulmonary hypertension, significant mitral regurgitation, mild tricuspid regurgitation, restrictive filling pattern (grade III), preserved right ventricular systolic function with impaired diastolic function, and mild pericardial effusion. Twenty-four-hour Holter monitoring showed a high burden of ventricular ectopy with frequent PVCs and multiple episodes of nonsustained ventricular tachycardia (VT). The patient was diagnosed with heart failure complicated by frequent ventricular arrhythmias and received continuous ECG monitoring and guideline-directed medical therapy. Bedside telemetry revealed polymorphic ventricular ectopy, short runs of VT, and incomplete left bundle branch block (Fig. 1B). Coronary angiography excluded significant coronary artery disease. In the absence of coronary lesions, arrhythmogenic cardiomyopathy (ACM) was suspected, and genetic testing was initiated.

**Figure 2.**
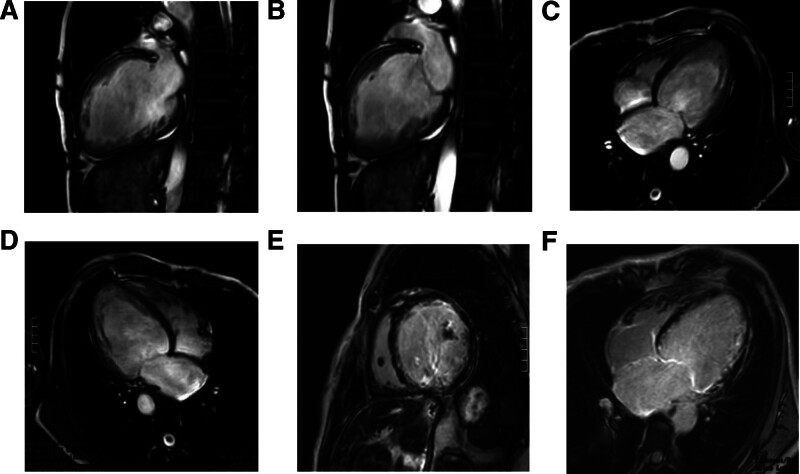
CMR demonstrating ALVC substrate and dyssynchrony with follow-up outcomes. Panels (A–F) illustrate an indistinct interface between the LV free wall and epicardial fat; diffuse LV free-wall thinning; marked enlargement of the left atrium and LV; severe global LV systolic dysfunction with mechanical dyssynchrony; and nonischemic subepicardial LGE of the LV free wall, consistent with ALVC. Coronary angiography (not shown) revealed no significant stenosis, supporting a nonischemic etiology. Baseline TTE LVEF was 19%; after CRT-D implantation, follow-up showed LVEF 27% at ~3 mo and 36% at ~6 mo, with no malignant ventricular arrhythmias on device interrogation. ALVC = arrhythmogenic left ventricular cardiomyopathy, CMR = cardiac magnetic resonance, CRT-D, cardiac resynchronization therapy–defibrillator, LGE = late gadolinium enhancement, LV = left ventricle, LVEF = left ventricular ejection fraction.

Cardiac magnetic resonance (CMR) imaging demonstrated diffuse thinning of the left ventricular free wall, severe global systolic dysfunction, impaired wall motion coordination, and nonischemic myocardial fibrosis, findings consistent with arrhythmogenic left ventricular cardiomyopathy (ALVC) (Fig. 2). Following discussion with the patient’s family, a cardiac resynchronization therapy defibrillator (CRT-D) was implanted. Postoperatively, recovery was uneventful, with stable vital signs and adequate device sensing on ECG monitoring. Genetic testing subsequently confirmed the diagnosis of ALVC.

The patient was discharged in stable condition on June 19, 2023. Ten days later, he was readmitted with pain and erythema at the pacemaker pocket site, consistent with a localized infection. Surgical debridement with negative-pressure wound therapy was performed under local anesthesia, and wound cultures grew fungal spores and Gram-positive cocci. Intravenous vancomycin (1000 mg every 12 hours) was administered for 1 week, resulting in resolution of drainage, and the patient was discharged in stable condition. At 3- and 6-month follow-up, left ventricular ejection fraction improved to 27% and 36%, respectively, with no ventricular arrhythmias detected on device interrogation.

## 3. Patient perspective

Before the diagnosis, I felt short of breath and tightness in my chest with simple activities like walking uphill. Rest helped for a few minutes, but the symptoms kept returning, and I fainted once, which frightened me and my family. During the hospital stay, I underwent many tests. Learning that I had arrhythmogenic left ventricular cardiomyopathy was stressful, but having a clear explanation of the condition and treatment plan helped me cope. After the CRT-D was implanted, I noticed gradual improvement in breathing and energy. Daily tasks such as walking and household chores became easier. Over the following months, I was able to resume light exercise and felt more confident, especially knowing that the device could treat dangerous rhythms if they occurred. Overall, I am grateful that the diagnosis was made and that the treatment improved my symptoms and daily life.

## 4. Discussion

ACM is defined as myocardial abnormalities that lead to arrhythmia independent of ischemia, hypertension, or valvular heart disease. Clinically, ACM may present with arrhythmias, heart failure, or atrial fibrillation, conduction disorders, or ventricular arrhythmias affecting either or both ventricles.^[7]^ This disease is relatively rare, with an estimated prevalence of 0.02% to 0.1%.^[8]^ ALVC, a subtype of ACM primarily involving the left ventricle, is characterized by progressive replacement of left ventricular myocardium with adipose and fibrous tissue, predisposing patients to fatal malignant arrhythmias and SCD.

ALVC affects a broad age range – from adolescence to over 80 years old)^[5]^ – and often presents with nonspecific symptoms such as chest tightness, dyspnea, or palpitations. Some patients initially manifest with ventricular arrhythmias, leading to syncope or SCD, especially among males, young individuals, and athletes.^[7,9]^ The first ALVC case diagnosed in Iran involved a 33-year-old male with a family history of SCD who presented with frequent PVCs on ECG, though heart failure was the initial symptom.^[10]^ Among patients with confirmed ALVC, 64% showed ventricular arrhythmias on ECG.^[11]^ A study of 60 ACM patients found that 25 initially presented with isolated arrhythmias such as palpitations, syncope, and/or VT, gradually progressing to heart failure or recurrent sustained VT, while the remaining 35 presented with heart failure with or without arrhythmias. The average age at symptom onset was 30.03 ± 11.74 years, and 83.33% eventually required heart transplantation due to heart failure with or without VT.^[12]^ Furthermore, studies on ACM phenotypes and clinical outcomes have shown that the incidence of heart failure in ALVC patients (6%) was higher than in ARVC patients (1%). Additionally, compared to ARVC patients (5%), hot-phase episodes (myocarditis-like symptoms, such as chest pain with elevated troponin and abnormal ECG despite normal coronary arteries) were more common in ALVC-affected patients (ALVC 14%).^[13]^ Intense exercise is a trigger for malignant arrhythmias, syncope, and SCD.^[9]^ In a large cohort study on SCD patients, 202 cases were associated with ACM, with 82% being male. Among them, 83 patients (41%) died during physical exertion, and 17% were diagnosed with ALVC,^[13]^ indicating that exercise increases ALVC incidence and SCD risk.^[14]^ Myocardial scarring has also been identified as a strong predictor of arrhythmic events in these patients.^[15]^ In our case, the patient’s symptoms of chest tightness, dyspnea, and syncope were associated with frequent PVCs and short bursts of VT on ECG, likely due to ALVC-related myocardial scarring leading to ventricular electrical instability.

The diagnosis of ALVC depends on the presence of cardiomyopathy originating mainly from the left ventricle in the proband or family members, not attributed to ischemic, valvular, or hypertensive heart disease. Although both echocardiography and CMR can detect left ventricular dysfunction or structural abnormalities, echocardiography alone often shows limited sensitivity and specificity in ALVC. In contrast, CMR provides superior myocardial tissue characterization, thereby improving diagnostic accuracy.^[5,16]^ The 2020 Padua diagnostic criteria highlight the need for CMR in myocardial tissue characterization and establish CMR as the primary diagnostic criterion for ALVC. Typical CMR findings include subepicardial late gadolinium enhancement or fibrosis located on the free wall of the left ventricle, with or without septal involvement.^[17]^ CMR is currently considered the “gold standard” for evaluating myocardial pathology, aiding in diagnosis, risk stratification, and treatment planning.^[18]^ It can also detect nonischemic ventricular scarring or myocardial tissue replacement by fat, combined with descriptions of ventricular dilation or systolic dysfunction, aiding in the prognosis of cardiomyopathy. In our case, CMR showed fibrosis on the epicardium, consistent with ALVC features. Differentiation from ischemic cardiomyopathy is necessary, with CAG providing confirmation. Frequent PVCs and bursts of VT led to left ventricular dysfunction, which reversed upon the elimination of these arrhythmias, supporting our diagnosis. Although endomyocardial biopsy confirmation of fatty tissue infiltration is the gold standard for ALVC, a negative biopsy does not rule out ALVC, and its risk/benefit ratio remains uncertain, making it not routinely used for ALVC diagnosis.

Anterior chest wall deformities – particularly pectus excavatum – should be considered because their mechanical impact on cardiac morphology and kinematics can produce a “cardiomyopathy-like” phenotype.^[19]^ Effects include RV compression and deformation, impaired filling, regional motion abnormalities, and ventricular arrhythmias, potentially confounding interpretation of TTE and speckle-tracking findings.^[19,20]^ On CMR and/or chest CT, some cases show focal retrosternal compression, cardiac levoposition, flattening/concavity and focal distortion of the RV anterior wall, and even “focal electrophysiologic cardiomyopathy” associated with PVCs/VT; surgical correction can improve RV volumes and biventricular systolic function.^[21]^ In contrast, LV-predominant ACM/ALVC typically demonstrates a nonischemic LGE pattern – subepicardial or mid-myocardial scar of the LV free wall – together with wall-motion abnormalities and/or systolic dysfunction, features that help distinguish it from pure mechanical compression.^[22]^ In our patient, careful review of chest radiography, TTE, and CMR revealed no focal RV compression or marked cardiac levoposition; conversely, CMR demonstrated subepicardial fibrosis and diffuse thinning of the LV free wall characteristic of ACM/ALVC, and genetic testing was positive – collectively supporting ALVC and effectively ruling out chest-wall-related functional impairment.

ALVC patients may face high risks of malignant arrhythmias, ventricular dysfunction, exercise intolerance, and SCD, which can affect long-term survival and prognosis. Thus, arrhythmia management, heart failure delay, and prophylactic implantable cardioverter-defibrillator (ICD) implantation are essential for ALVC patients. Catheter ablation is often recommended for patients with sustained VT. Studies indicate that ICD can reduce mortality in VT patients by 28%.^[23]^ However, ICD implantation does not prevent SCD and should not replace VT treatment.^[24,25]^ Ventricular involvement is strongly associated with heart failure, often manifesting left ventricular dysfunction even before heart failure diagnosis, with a poor prognosis. Medications such as angiotensin-converting enzyme inhibitors, angiotensin receptor blockers, and beta-blockers are recommended by guideline for heart failure management.^[4]^ For ALVC-diagnosed patients or family members undergoing screening, ICD implantation may be considered for primary prevention, though large-scale clinical studies are needed to confirm survival benefit. In this case, the patient presented with LV-dominant arrhythmogenic cardiomyopathy complicated by severe systolic dysfunction, electrical dyssynchrony, and a high burden of ventricular arrhythmias, thereby fulfilling standard guideline-based indications for CRT-D implantation. Under guideline-directed medical therapy combined with CRT-D, the patient demonstrated progressive left ventricular functional recovery and absence of malignant ventricular arrhythmias during follow-up. According to the ESC 2023 cardiomyopathy guidelines,^[25]^ ICD therapy is indicated for secondary prevention and for primary prevention in selected high-risk patients after multiparametric risk assessment, whereas CRT follows standard heart-failure criteria (e.g., LVEF ≤35% with electrical/mechanical dyssynchrony and persistent symptoms despite optimized GDMT), independent of the specific cardiomyopathy subtype. In LV-dominant ACM/ALVC with dyssynchrony, CRT can be combined with defibrillator protection as CRT-D to address both reverse remodeling and sudden cardiac death prevention.

## 5. Conclusion

In conclusion, ALVC is a rare cardiac condition with diverse clinical presentations, including high rates of ventricular arrhythmias and left ventricular fibrosis before myocardial injury and systolic dysfunction. In patients with suspected ischemic symptoms but no significant coronary artery lesions on angiography, CMR should be considered to aid in diagnosis. Risk assessments for family members is also recommended, focusing on early cardiovascular events (such as SCD, heart failure) and related cardiac phenotypes (such as arrhythmias and conduction disorders). Early identification and timely intervention are essential for improving prognosis and preventing adverse outcomes.

## Author contributions

**Data curation:** Xinhe Cheng, Yang Liu.

**Formal analysis:** Xinhe Cheng.

**Investigation:** Xin Wang.

**Methodology:** Xinhe Cheng, Xin Wang.

**Project administration:** Xin Wang.

**Supervision:** Yang Liu.

**Writing – original draft:** Xin Wang, Yang Liu.

**Writing – review & editing:** Xin Wang, Yang Liu.

## Supplementary Material


