# Supplementary material for: Arrhythmogenic left ventricular cardiomyopathy managed with CRT-D: A case report
Source: Medicine (Baltimore). 2026 Mar 6;105(10):e47481. doi: 10.1097/MD.0000000000047481 (PMC12975253; doi:10.1097/MD.0000000000047481)
Supplement: Supplementary file 1 [file medi-105-e47481-s001.docx]

Supplementary Table 1. Timeline of treatment details.

| Date(s) | Medication / Procedure | Dose & Administration | Route / Frequency | Indication / Notes |
| --- | --- | --- | --- | --- |
| Jun 1–8 | Aspirin enteric-coated | 0.1 g | PO, QN | Antiplatelet while ischemia under evaluation |
| Jun 1–8 | Rosuvastatin | 10 mg | PO, QN | Lipid lowering / plaque stabilization |
| Jun 1–8 | Furosemide (tablet) | 20 mg | PO, QD | Decongestion/diuresis |
| Jun 1–8 | Spironolactone | 20 mg | PO, QD | MRA for HF/anti-remodeling |
| Jun 1–8 | Dapagliflozin | 10 mg | PO, QD | SGLT2 inhibitor for HF benefit/diuresis |
| Jun 1–8 | Digoxin | 0.125 mg | PO, QD | Positive inotropy / rate support (monitor levels) |
| 2-Jun | Metoprolol succinate (ER) | 23.75 mg | PO, QD | β-blocker initiation |
| Jun 2–6 | Levosimendan | 12.5 mg, 1 mL/h | Inf (pump) | Calcium-sensitizer; inotrope for low output |
| Jun 2–13 | Shensong Yangxin capsule | 1.2 g | PO, QD | Adjunct symptom control (TCM) |
| 2-Jun | Potassium chloride “injection” (given orally) | 10 mL | PO, ST | Potassium repletion |
| 2-Jun | Glutamine & sodium gualenate granules | 0.67 g | PO, ST | Nutritional/metabolic support |
| Jun 3–15 | Coenzyme Q10 capsule | 10 mg | PO, daily | Metabolic support/antioxidant |
| 4-Jun | 0.9% NaCl 250 mL + 10% KCl 7 mL + magnesium sulfate 2.5 g | — | IV, infusion | Electrolyte repletion (K/Mg) |
| Jun 5–7 | Recombinant human B-type natriuretic peptide | 0.5 mg, 4 mL/h | Inf (pump) | Vasodilation/diuresis; decongestion |
| 6-Jun | Furosemide (injection) | 20 mg | IVP, ST | Intensified diuresis |
| 7-Jun | Ticagrelor | 180 mg | PO, ST (loading) | Antiplatelet loading (periprocedural) |
| 7-Jun | Furosemide (injection) | 20 mg | IVP, ST | Intensified diuresis |
| 8-Jun | Coronary angiography | — | Procedure, ST | No significant stenosis (ischemia excluded) |
| Jun 8 (intra-op) | 0.9% NaCl | 500 mL | IV, infusion, ST | Peri-procedural fluids |
| Jun 15 (12:00 & 18:00) | Cefazolin (pre-op prophylaxis) | 2 g each | IV, ST ×2 | CIED surgical prophylaxis (gram-positive coverage) |
| Jun 15, 19:00 | CRT-D implantation | — | Procedure, ST | Device therapy for dyssynchrony/arrhythmia risk |
| Jun 15–19 | Sacubitril/valsartan | 25 mg | PO, BID | ARNI initiation for HFrEF/anti-remodeling |
| Jun 15–19 | Metoprolol succinate (ER) | 47.5 mg | PO, QD | β-blocker uptitration |
| 15-Jun | Iodinated contrast agent (370 mg I/mL) | 50 mL | IV, ST | Peri-procedural imaging |
| 19-Jun | Hospital discharge | — | — | Stable, on guideline-directed therapy |
